# Survey of socio-economic and contextual factors of households׳ energy consumption

**DOI:** 10.1016/j.dib.2015.09.007

**Published:** 2015-09-26

**Authors:** Omar Jridi, Fethi Zouheir Nouri

**Affiliations:** a Laboratory of Research in Applied Microeconomics (LARMA), Faculty of Economic Sciences and Management, Tunis ELMANAR University, Campus University, B.P. 248-El Manar II-2092, Tunisia; b High School of Economic Sciences and Commercials, Tunis University, LARMA, 4, Abou Zakaria El Hafsi Street, 1089 Montfleury, Tunisia

**Keywords:** Buildings, Households, Energy saving equipment, Survey

## Abstract

We present a set of data relating to the investigation of the Tunisian Company of Electricity and Gas (STEG). The census is done on a sample of 3000 electrified households. The questionnaire is divided into three main sections: household socioeconomic status, contextual characteristics related to their housing and technical characteristics of equipments used. The objective of this survey is to achieve a reliable and detailed knowledge on the behavior of household energy consumption, particularly for energy saving behavior. This objective has recently been the subject of a research article Jridi et al. (2015) [2].

## 1 Specifications Table

**Table:** 

Subject area	Economics
More specific subject area	Buildings, households, energy saving equipments
Type of data	Table, figure
How data was acquired	Survey
Data format	Raw, analyzed
Experimental factors	Geographical and socio-economic stratification
Experimental features	Equipments are classified according to their energy efficiencies. The adoption of energy saving equipment is essentially explained by the characteristics of households, Buildings and equipments.
Data source location	All governorates of Tunisia
Data accessibility	Descriptive analysis of data is provided in this article and raw data of the Tunisian Company of Electricity and Gas (STEG) is presented in supporting information.

## 2 Value of the data

● Bring a deep knowledge of the end-use of residential energy.
● Knowing the behavior, opinions and projects household on energy choices and corresponding equipments.
● Identify the impact of certain socio-economic and geographic variables on the nature of the equipment and on residential energy consumption.
● Future research on the behavior of energy use will be facilitated by the data included here.

## 3 Data, experimental design, materials and methods

### 3.1 Data

Since 1984, the Tunisian Company of Electricity and Gas is committed to making quinquennial census surveys about the energy use of its residential customers [1]. In this article, we present the latest survey data received from 3000 households. Sampling methodology is based on the principles of socio-economic and geographical stratification and random selection. The response rate is 96%, of which 92.9% are deemed correct answers. The questionnaire is divided into three sections: (i) the socioeconomic status of the household (age, activity, income, educational level, etc.). (ii) Housing (dwelling type, tenure status, date of construction, number of parts, etc.). (iii) The residential energy equipments, of which STEG gives attention to the energy saving equipments, namely energy saving lamps, the solar water heaters, labeling of refrigerators [2].

**Fig. 1:**
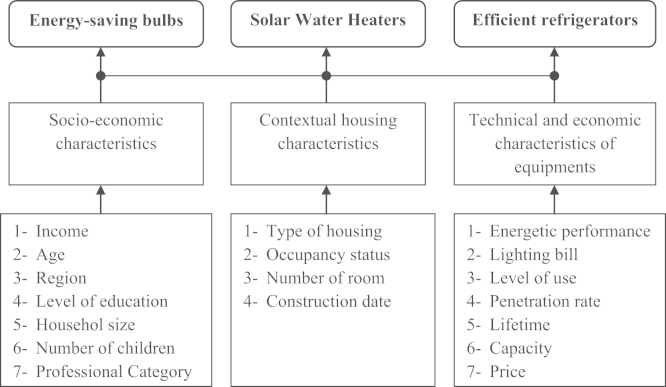
Structure of the database.

The objective of this survey is to identify the determinants of the adoption of the energy saving equipments. We consider three electrical purposes: water heaters solar, efficient refrigerators and energy saving lamps. The determining factors are classified in three categories: socio-economic characteristics of households, buildings characteristics and the technical and economic characteristics of equipments (see Fig. 1).

### 3.2 Materials and methods for the case of water heater

The first energy saving measure promotes the purchase of solar water heaters as an alternative to other types of water heaters that exist on the market (electric, natural gas and LPG) [4]. In addition to explanatory factors identified above, we include a dummy variable “Dummy for connection to the natural gas network.” This variable takes into account the effect of the strategy adopted by Tunisia concerning the natural gas connection on the adoption of solar water heaters. Table 1 shows these descriptive statistics of each type water heaters. With *h*_1_ explanatory variables, identifying the weight of the various factors through the following equation:

$$\ln\left(\frac{P\left(\mathrm{WH}=\mathrm{solar}\right)}{P\left(\mathrm{WH}=\mathrm{auther}\right)}\right)=\;\beta_{0}+\;\sum_{i=1}^{h_{1}}\beta_{i}\;X_{i}$$

### 3.3 Materials and methods for the case of energy class refrigerators

The second energy saving measure relates to refrigerators with efficient energy classes. With the coming into force of refrigerators labeling program, which prohibits the marketing of refrigerators without energy label, it seems insignificant to take into account households that have refrigerators without energy classes. So we extract the sample of households that have refrigerators with energy labels, we get 1616 households having refrigerators with energy classes from 1 to 8. To do this we incorporate a dummy variable "dummy for certification", which takes into account the effect of the entry in strengths of refrigerators certification program. If the age of the refrigerator does not exceed five years, the dummy variable takes the value 1 (it is set to 0 if not) (Table 2).

At this level, to form coalitions with the prospects of the certification program of refrigerators, which provides, from 2015, the elimination of the least than class 2 efficient energy classes, we assume that refrigerators incorporate this category in one energy class, that we call non-performing class “NP class”. This class is defined as the reference alternative. This choice is explained by two categories of explanatory variables: the technical characteristics of refrigerators (capacity, in liters and energy requirement, in kilowatt) and socio-economic characteristics of the household (such as income, utility bill, number of months in the refrigerator connection, etc.) [3,5]. With *h*_2_ explanatory variables, identifying the weight of the various factors on the choice of the classes 1 and 2 through by the following two equations:

$$\ln\left(\frac{P\left(\mathrm{energy}\;\mathrm{class}=1\right)}{P\left(\mathrm{energy}\;\mathrm{class}=\mathrm{NP}\right)}\right)=\;\beta_{10}+\;\sum_{i=1}^{h_{2}}\beta_{1i}\;X_{i}$$

$$\ln\left(\frac{P\left(\mathrm{energy}\;\mathrm{class}=2\right)}{P\left(\mathrm{energy}\;\mathrm{class}=\mathrm{NP}\right)}\right)=\;\beta_{20}+\;\sum_{i=1}^{h_{2}}\beta_{2i}\;X_{i}$$

### 3.4 Materials and methods for the case of energy saving lamps

**Fig. 2:**
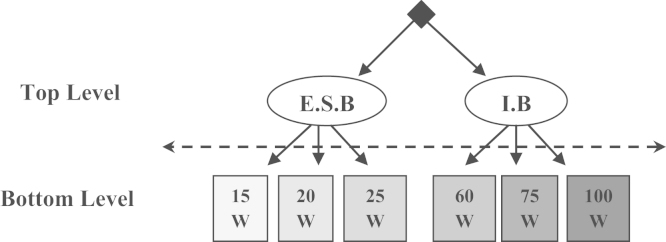
Tree structure of levels choice׳s of lighting lamps.

Regarding the illumination station, and to focus attention on promoting energy saving lamps, we assume that the choice of households is done at two levels, without imposing a sequential order in the choice. The top level when the household chooses between incandescent lamps (IL) and energy saving lamps (ESL). The Bottom level where the household chooses the lamp power level (expressed in watts). Fig. 2 shows these levels and the possible choices.

Similar to the tree structure of Fig. 2, the choice in the top level is supposed to be explained by socio-economic characteristics of households (income, household size, number of rooms and lighted area of residence) [3]. The choice in the Bottom level is supposed to be explained by the price and the lifetime of each bulb, as well as the conventional lighting bill attributable to the common use of household and level the capacity of the bulb used [6]. Table 3 shows these descriptive statistics by level of possible choice.

## Conflict of interest

None.

## Figures and Tables

**Table 1:** Descriptive statistics for water heater by energy source requested.

***Attributes***	**Electro-solar**	**Electric**	**LPG (bottled)**	**STEG gas**	**Total**
**Housing characteristics**					
Occupancy status					
Tenant (%)	26	9.68	17.95	23.36	19.21
Owner (%)	74	90.32	82.05	76.64	80.79
Type of housing					
Appartment (%)	14	6.45	9.1	32.71	13.81
Traditional (%)	16	17.74	23.45	7.94	19.65
Popular (%)	25	19.35	19.35	21.96	30.74
Villa (%)	45	56.45	56.45	37.38	37.81
**Household characteristics**					
Income (Tunisian dinar)	1084	510.96	556.55	764.73	636.05
Mean household size	4.16	4.48	4.49	4.07	4.38
Region					
Communal (%)	79	75.81	85.18	94.86	85.94
Rural (%)	21	24.19	14.82	5.14	14.06
Level of education					
Illiterate (%)	10	9.68	11.05	10.28	10.74
Primary (%)	10	20.97	24.84	14.49	21.4
Secondary (undergraduate) (%)	19	19.35	23.54	16.82	21.66
Secondary (second cycle) (%)	21	24.19	23.67	28.97	24.45
Faculty (%)	40	25.81	16.91	29.44	21.75
**Dummy for connection to the natural gas network (%)**	5.65	3.18	19.79	71.38	24.72
**Total observation**	**100**	**62**	**769**	**214**	**1145**

**Table 2:** Descriptive statistics for energy class refrigerators.

***Attributes***	**Energy class 1**	**Energy class 2**	**Energy class NP**	**Total**
**Technical characteristics**				
Capacity (L)	255	253	246–235	237
Energy needs (kW h/year)	302	356	438−498	392
**Household** characteristics				
Number of refrigerator	1.13	1.03	1.04	1.05
Month of connection	10.07	9.77	9.35	9.36
Average electricity consumption (kW h)	1617.3	1628.3	1344.5	1433.9
Income (Tunisian dinar)	621.7	785.5	379.4	497.9
Region				
Communal (%)	88.24	80.54	63.19	69.18
Rural (%)	11.79	19.46	36.81	30.82
Level of education				
Illiterate (%)	11.76	3.56	23.66	17.82
Primary (%)	22.55	7.3	39.89	30.51
Secondary (undergraduate) (%)	13.73	3.41	22.85	17.33
Secondary (second cycle) (%)	32.35	46.72	7.07	18.75
Faculty (%)	19.61	38.93	6.53	15.59
**Total observation**	**102**	**411**	**1103**	**1616**

**Table 3:** Descriptive Statistics for Lighting Park by bulb power.

	15 W	20 W	25 W	60 W	75 W	100 W	Total
***Attributes***							
Income (TD)	666.2	568.8	604.1	462.5	409.6	384.9	478.6
Mean number of children	1.28	1.53	1.39	1.39	1.50	1.16	1.42
Average lighted room	6.20	5.91	5.64	4.69	4.79	4.61	5.11
**Region**							
Communal (%)	85.78	80.17	84.08	59.49	61.24	64.47	68.27
Urban (%)	14.22	19.83	15.92	40.51	38.76	35.53	31.73
Power bulb (W)	15	20	25	60	75	100	–
Average utilization (hours/day)	3.90	4.48	4.06	2.50	2.88	2.76	3.23
Lighting bill (TD)	0.63	0.95	1.08	1.61	2.31	2.96	1.83
Average number of bulbs	6.83	7.19	5.97	4.49	4.73	4.35	5.24
Price (TD)	14	9	8	1.4	1	0.8	3.71
**Total observation**	**232**	**233**	**314**	**352**	**1112**	**228**	**2 471**
